# Longitudinal Myocardial Deformation as an Emerging Biomarker for Post-Traumatic Cardiac Dysfunction

**DOI:** 10.3390/life15071052

**Published:** 2025-06-30

**Authors:** Makhabbat Bekbossynova, Timur Saliev, Murat Mukarov, Madina Sugralimova, Arman Batpen, Anar Kozhakhmetova, Zhumagul Sholdanova

**Affiliations:** 1 Heart Center, “University Medical Center” Corporate Fund, Nazarbayev University, Astana 010000, Kazakhstan; m.bekbosynova@umc.org.kz (M.B.);; 2 Institute of Fundamental and Applied Medical Research, S.D. Asfendiyarov Kazakh National Medical University, Tole Bi Street 94, Almaty 050000, Kazakhstan; 3 National Scientific Center of Traumatology and Orthopedics Named After Academician N.D. Batpenov, Astana 010000, Kazakhstan

**Keywords:** cardiac dysfunction, global longitudinal strain, speckle-tracking echocardiography, myocardial injury, polytrauma, cardiac biomarkers

## Abstract

Post-traumatic cardiac dysfunction is a clinically under-recognized complication of polytrauma, often occurring in the absence of overt structural injury. Traditional diagnostic tools frequently fail to detect early or subclinical myocardial impairment, underscoring the need for more sensitive assessment methods. This review explores the utility of global longitudinal strain (GLS), derived from speckle-tracking echocardiography (STE), as a sensitive biomarker for identifying and managing cardiac dysfunction following traumatic injury. It outlines the complex pathophysiology of trauma-induced myocardial impairment, including mechanical injury, systemic inflammation, oxidative stress, and neuro-hormonal activation. The limitations of conventional diagnostic approaches, such as electrocardiography, left ventricular ejection fraction (LVEF), and cardiac biomarkers, are critically assessed and contrasted with the enhanced diagnostic performance of GLS. GLS has demonstrated superior sensitivity in detecting subclinical myocardial dysfunction even when LVEF remains preserved and is associated with increased risk of long-term cardiovascular complications, including arrhythmias and heart failure. The manuscript highlights the clinical utility of GLS in early diagnosis, risk stratification, treatment monitoring, and long-term follow-up. Integration of GLS with inflammatory and oxidative biomarkers (e.g., IL-6, TNF-α, and MPO) and artificial intelligence-based diagnostic models offers potential for improved precision in trauma cardiology.

## 1. Introduction

Post-traumatic cardiac dysfunction is an under-recognized yet critical consequence of severe trauma, often overlooked in the acute management of polytrauma patients. While the primary focus in trauma care is on immediate life-threatening injuries, subtle myocardial impairment can develop due to direct cardiac contusion, systemic inflammation, neurohormonal dysregulation, and oxidative stress [1]. Traditional diagnostic tools, including electrocardiography (ECG), cardiac biomarkers, and conventional echocardiography, frequently lack the sensitivity to detect early or subclinical myocardial dysfunction, leading to missed opportunities for timely intervention [2,3,4]. Given the increasing recognition of cardiovascular complications following trauma, there is a growing need for more precise diagnostic methods to assess myocardial injury and guide clinical management [5].

Although the exact prevalence of subclinical myocardial dysfunction following trauma is underreported due to diagnostic limitations, data from intensive care settings suggest that approximately 25–30% of critically ill patients exhibit impaired myocardial strain despite preserved ejection fraction [6]. This prospective cohort study evaluated left ventricular global longitudinal strain (LV GLS) using speckle-tracking echocardiography in 152 normotensive septic ICU patients [6]. Non-survivors had worse LV GLS (−15.2 vs. −17.3, *p* < 0.001) and a cut-off of −17% predicted ICU mortality (AUC 0.728). Patients with LV GLS >−17% had higher mortality (39.2% vs. 13.7%, *p* < 0.001). Multivariate analysis confirmed LV GLS as an independent mortality predictor (OR 1.326, *p* = 0.024), alongside mechanical ventilation and severity scores. Impaired LV GLS is linked to higher mortality in normotensive septic patients. Similarly, among septic patients with normal LVEF, GLS ≥ −13% was independently predictive of ICU and hospital mortality (HR ~4.3), even when troponin and LF-EF were unremarkable [7]. These findings underscore the clinical importance of incorporating strain imaging into early trauma assessment as GLS abnormalities frequently precede overt symptoms and traditional diagnostic markers, offering a crucial window for intervention.

Longitudinal myocardial deformation, assessed through speckle-tracking echocardiography (STE), has emerged as a promising tool in the evaluation of post-traumatic cardiac dysfunction. Unlike left ventricular ejection fraction (LVEF), which reflects global ventricular function and may remain preserved in early myocardial impairment, global longitudinal strain (GLS) provides a more detailed and quantitative measure of myocardial contractility. GLS has been shown to detect subclinical myocardial dysfunction in various cardiac conditions, including heart failure, myocardial infarction, and cardiomyopathies, making it an ideal candidate for trauma-related myocardial assessment [8,9]. The application of GLS in trauma care holds significant promise for improving early detection, monitoring myocardial recovery, and guiding treatment strategies. Trauma patients with preserved LVEF but impaired GLS may be at higher risk for long-term cardiovascular complications, necessitating closer cardiac surveillance and potential pharmacologic interventions [10,11]. Additionally, GLS has been proposed as a valuable tool for evaluating treatment response as improvements in myocardial strain over time can provide insights into cardiac recovery and therapeutic efficacy.

GLS was selected for its superior sensitivity in detecting subclinical myocardial dysfunction compared to traditional measures such as left ventricular ejection fraction (LVEF), tissue Doppler imaging (TDI), or cardiac biomarkers. Unlike LVEF, which often remains preserved in early myocardial injury, GLS can quantify subtle changes in myocardial fiber deformation before overt systolic dysfunction develops. Additionally, GLS is angle-independent, and reproducible, and can be measured via standard 2D speckle-tracking echocardiography, making it a feasible and non-invasive tool in the acute trauma setting.

Despite its advantages, the clinical implementation of GLS in trauma settings faces several challenges. Variability in strain measurement techniques across different echocardiographic platforms, the need for specialized imaging expertise, and the lack of established reference values for trauma patients limit the widespread adoption of this technique [10,12]. Further research is needed to validate GLS as a reliable biomarker for post-traumatic cardiac dysfunction and to establish standardized protocols for its use in trauma populations. Moreover, integrating GLS with complementary diagnostic tools, such as cardiac biomarkers and emerging artificial intelligence (AI)-driven imaging analytics, could enhance its predictive value and clinical applicability [13,14].

Traditional diagnostic tools such as left ventricular ejection fraction (LVEF) and standard electrocardiography (ECG) often fail to detect subtle myocardial dysfunction in the early post-trauma phase. For instance, several studies have reported that trauma patients with normal LVEF on echocardiography exhibited significantly reduced global longitudinal strain (GLS), indicating occult systolic impairment [15,16]. In these cases, GLS abnormalities preceded any decline in ejection fraction or visible ECG changes, underscoring its sensitivity for identifying early myocardial injury that would otherwise remain undiagnosed.

Trauma-induced myocardial dysfunction is multifactorial, involving both direct mechanical injury and systemic physiological responses. Blunt chest trauma, often resulting from motor vehicle accidents, falls, or physical impact, can cause myocardial contusion, leading to localized ischemia, oedema, and wall motion abnormalities [17,18]. However, myocardial impairment is not limited to direct injury. Trauma triggers a systemic inflammatory response, characterized by the release of cytokines such as interleukin-6 (IL-6) and tumor necrosis factor-alpha (TNF-α), which contribute to endothelial dysfunction, increased vascular permeability, and myocardial inflammation [19]. Oxidative stress and neurohormonal activation further exacerbate myocardial damage, leading to myocardial stunning, impaired contractility, and potential long-term cardiovascular complications [20,21]. These complex pathophysiological mechanisms underscore the need for advanced diagnostic approaches that can accurately capture myocardial dysfunction beyond traditional methods.

This narrative review aims to explore the role of longitudinal myocardial deformation as an emerging biomarker for post-traumatic cardiac dysfunction. It examines the pathophysiological mechanisms underlying myocardial impairment in trauma patients, the limitations of traditional diagnostic methods, and the potential advantages of GLS in detecting subclinical myocardial dysfunction. Furthermore, the review discusses the clinical applications of GLS in trauma care, including its role in early diagnosis, risk stratification, treatment monitoring, and long-term follow-up. Future research directions, including the integration of AI in strain analysis and the development of standardized strain assessment protocols, are also highlighted.

## 2. Pathophysiology of Post-Traumatic Cardiac Dysfunction

Post-traumatic cardiac dysfunction is a complex and multifaceted condition that arises from a combination of direct myocardial injury, systemic inflammation, oxidative stress, neurohormonal activation, and immune dysregulation [22]. Unlike ischemic heart disease, where coronary artery occlusion is the primary insult, cardiac dysfunction following trauma results from a cascade of pathophysiological events that impair myocardial function and increase long-term cardiovascular risk. Trauma-induced myocardial dysfunction can occur even in the absence of direct cardiac injury, making it a challenging condition to diagnose and manage [23]. Understanding the intricate interplay between inflammatory, oxidative, and neurohormonal pathways is crucial for identifying high-risk patients and developing targeted therapeutic interventions.

Blunt chest trauma is one of the most direct causes of myocardial dysfunction in trauma patients [24]. High-impact injuries, such as those sustained in motor vehicle accidents or falls, can lead to myocardial contusion, hemorrhage, and necrosis. These localized effects contribute to regional wall motion abnormalities, impaired contractility, arrhythmias, and conduction disturbances. In severe cases, myocardial rupture or cardiac tamponade can occur, leading to hemodynamic instability and life-threatening consequences [25]. Even when structural damage is not apparent, mechanical stress can lead to subcellular injury, calcium dysregulation, and metabolic disturbances that compromise cardiac function. However, beyond mechanical trauma, systemic factors play a crucial role in the development of cardiac dysfunction.

A systemic inflammatory response following severe injury is a major driver of trauma-induced cardiac dysfunction. Polytrauma triggers a massive immune response characterized by the release of pro-inflammatory cytokines, including interleukin-6 (IL-6), tumor necrosis factor-alpha (TNF-α), and interleukin-1 beta (IL-1β) [26]. These cytokines contribute to endothelial activation, increased vascular permeability, and leukocyte infiltration into the myocardium, leading to myocardial oedema and contractile dysfunction. Elevated levels of IL-6 correlate with increased myocardial injury and worse clinical outcomes, while TNF-α and IL-1β have been implicated in endothelial dysfunction, smooth muscle proliferation, and cardiac fibrosis [27]. Persistent immune activation exacerbates myocardial stress and increases the risk of long-term complications such as heart failure and arrhythmias.

Oxidative stress further amplifies myocardial injury in trauma patients. The excessive generation of reactive oxygen species (ROS) due to ischemia–reperfusion injury, hypoxia, and excessive neutrophil activation results in mitochondrial dysfunction, lipid peroxidation, and cardiomyocyte apoptosis [28]. Oxidative damage is exacerbated by myeloperoxidase (MPO), a pro-oxidant enzyme released by neutrophils, which contributes to endothelial damage, microvascular thrombosis, and nitric oxide depletion [29]. The cumulative effects of oxidative stress impair myocardial energy metabolism, reduce contractility, and promote fibrosis. These changes not only worsen acute cardiac dysfunction but also set the stage for chronic myocardial remodeling, increasing the risk of diastolic dysfunction and heart failure.

The neurohormonal response to trauma plays a significant role in cardiac dysfunction. Following severe injury, the sympathetic nervous system is activated, leading to a surge in catecholamines such as epinephrine and norepinephrine [30,31]. While this response is initially adaptive, prolonged sympathetic overactivity places excessive stress on the myocardium. Increased heart rate, contractility, and vascular resistance result in heightened myocardial oxygen demand, potentially leading to ischemia and cardiomyocyte injury. Catecholamine excess also disrupts calcium homeostasis, triggering hypercontractility, arrhythmias, and cellular apoptosis. In extreme cases, this neurohormonal stress response manifests as stress cardiomyopathy (Takotsubo cardiomyopathy), characterized by transient left ventricular dysfunction and wall motion abnormalities [32,33].

Endothelial dysfunction and microvascular thrombosis contribute to impaired myocardial perfusion and exacerbate cardiac injury. The inflammatory response following trauma promotes the upregulation of adhesion molecules such as intercellular adhesion molecule-1 (ICAM-1) and vascular cell adhesion molecule-1 (VCAM-1), facilitating monocyte and neutrophil adhesion to the endothelium [34]. This process leads to increased vascular inflammation, microvascular dysfunction, and reduced nitric oxide bioavailability, impairing vasodilation and myocardial perfusion. In addition, platelet activation and increased thromboxane A2 (TXA2) levels promote clot formation, increasing the risk of microvascular thrombosis and ischemia [35]. These factors create a self-perpetuating cycle of inflammation, vascular dysfunction, and cardiac stress that worsens myocardial injury over time.

As the inflammatory and oxidative cascades progress, ventricular remodeling and fibrosis develop, leading to long-term structural and functional alterations in the heart. The persistent activation of fibroblasts and excessive collagen deposition stiffen the myocardium, impairing relaxation and reducing compliance [36]. Over time, this process contributes to diastolic dysfunction, increased left ventricular filling pressures, and eventual heart failure. Patients who experience post-traumatic cardiac dysfunction are at a heightened risk of developing chronic cardiovascular diseases, including hypertensive heart disease, arrhythmias, and thromboembolic complications [37]. Therefore, early recognition and intervention are critical to improving long-term cardiac outcomes.

Given the complex and multifactorial nature of trauma-induced cardiac dysfunction, early identification of high-risk patients is essential. Conventional diagnostic tools such as electrocardiography (ECG) and standard echocardiography may not adequately detect subtle myocardial dysfunction. Advanced imaging modalities, including speckle-tracking echocardiography (STE) and cardiac MRI, provide more detailed assessments of myocardial mechanics, allowing for earlier detection of contractile abnormalities [38]. In addition, biomarker profiling, including IL-6, TNF-α, MPO, and ICAM-1, may offer valuable insights into the inflammatory and oxidative pathways driving myocardial dysfunction. Integrating these biomarkers into clinical practice may help refine risk stratification and guide targeted treatment approaches.

Anti-inflammatory agents such as IL-1β inhibitors (e.g., canakinumab) have demonstrated potential in reducing cardiovascular events in patients with chronic inflammation and may offer benefits in trauma-related cardiac dysfunction [39]. Similarly, antioxidant therapies targeting ROS and MPO may help reduce oxidative damage and preserve myocardial function [40]. In addition, interventions that modulate the neurohormonal response, such as beta-blockers, may help mitigate catecholamine-induced myocardial stress and prevent long-term dysfunction [41].

The pathophysiology of post-traumatic cardiac dysfunction is a complex interplay of mechanical, inflammatory, oxidative, and neurohormonal factors that contribute to acute and chronic myocardial impairment. Understanding these mechanisms is critical for improving early detection, risk stratification, and treatment strategies in trauma patients. By integrating advanced imaging techniques, biomarker profiling, and targeted therapeutic interventions, clinicians can better manage trauma-induced cardiac dysfunction and improve long-term cardiovascular outcomes.

## 3. Limitations of Traditional Diagnostic Methods in Trauma-Related Cardiac Dysfunction

The diagnosis of trauma-related cardiac dysfunction remains a significant challenge due to the complex interplay of mechanical injury, systemic inflammation, oxidative stress, and neurohormonal activation. While traditional diagnostic tools such as electrocardiography (ECG), conventional echocardiography, and cardiac biomarkers provide valuable information, they often fail to detect subtle myocardial dysfunction, leading to delays in diagnosis and treatment [42]. Many cases of post-traumatic myocardial impairment occur without overt structural damage, making it difficult to identify at-risk patients using standard diagnostic approaches. The limitations of traditional diagnostic methods underscore the need for more advanced, sensitive techniques that can detect early myocardial changes and guide timely intervention (Table 1).

Electrocardiography (ECG) is widely used in the initial evaluation of trauma patients with suspected cardiac involvement. It is useful for detecting arrhythmias, conduction abnormalities, and ischemic changes that may indicate myocardial injury. However, ECG has several limitations in the context of trauma-related cardiac dysfunction. Many trauma patients present with non-specific ECG findings that do not provide definitive evidence of myocardial impairment [43]. Transient ST-segment and T-wave abnormalities are common but lack specificity for diagnosing myocardial contusion or inflammation. In addition, ECG changes often do not correlate with the severity of myocardial dysfunction, and some patients with significant trauma-induced cardiac injury may have normal ECG findings. The reliance on ECG alone can lead to underdiagnosis of subtle myocardial dysfunction, particularly in cases where structural damage is not apparent (Table 1).

Conventional echocardiography is another key tool for assessing cardiac function in trauma patients. It provides real-time imaging of ventricular contractility, wall motion abnormalities, and pericardial effusion, which are critical for diagnosing myocardial contusion, cardiac tamponade, and other forms of post-traumatic cardiac dysfunction [44]. However, traditional echocardiographic parameters, such as left ventricular ejection fraction (LVEF), have limitations in detecting early myocardial dysfunction. LVEF is a measure of systolic function that reflects the percentage of blood ejected from the left ventricle during each contraction [45]. While a reduced LVEF is indicative of severe cardiac impairment, many trauma patients with myocardial dysfunction have a preserved ejection fraction, masking underlying contractile abnormalities. Standard echocardiographic techniques may not be sensitive enough to detect subtle myocardial strain, which can be an early sign of trauma-induced myocardial impairment (Table 1).

Another limitation of conventional echocardiography is its inability to provide detailed insights into myocardial mechanics. Speckle-tracking echocardiography (STE) and strain imaging have emerged as more advanced tools that can assess myocardial deformation, but these techniques are not yet widely used in routine trauma care [38]. Strain analysis provides a quantitative assessment of myocardial contractility by measuring the percentage of myocardial shortening during systole. GLS, in particular, has been shown to be a more sensitive marker of myocardial dysfunction than LVEF [46]. Studies have demonstrated that trauma patients with preserved LVEF but impaired GLS are at higher risk of developing long-term cardiovascular complications [47]. The limited availability of strain imaging in trauma settings represents a significant gap in current diagnostic capabilities.

Cardiac biomarkers, including high-sensitivity troponin (hs-Tn) and brain natriuretic peptide (BNP), play a crucial role in diagnosing myocardial injury [48] (Table 1). Troponin is a highly specific marker of myocardial cell damage and is widely used in the evaluation of suspected myocardial infarction [49]. In trauma patients, elevated troponin levels may indicate myocardial contusion, ischemia, or stress cardiomyopathy [50]. However, troponin elevation alone is not always indicative of direct cardiac injury as trauma-related factors such as systemic inflammation, hypoxia, and catecholamine surge can also contribute to elevated troponin levels [51]. This makes it challenging to distinguish between primary myocardial injury and secondary myocardial stress responses. Additionally, some trauma patients with significant myocardial dysfunction may have normal troponin levels, particularly if the damage is more functional than structural.

Brain natriuretic peptide (BNP) and N-terminal pro-brain natriuretic peptide (NT-proBNP) are markers of cardiac stress and heart failure that have been investigated in trauma patients [52,53]. Elevated BNP levels may reflect increased ventricular wall stress due to fluid overload, pulmonary hypertension, or myocardial dysfunction. While BNP measurements can provide valuable insights into cardiac status, they are non-specific and may be elevated due to non-cardiac factors such as renal dysfunction, sepsis, or severe trauma-related inflammation [54,55]. The lack of specificity of BNP and troponin limits their ability to serve as standalone diagnostic markers for trauma-related cardiac dysfunction.

Computed tomography (CT) and magnetic resonance imaging (MRI) are additional imaging modalities used in the evaluation of trauma patients [56,57]. CT angiography is highly effective in detecting aortic injuries, pericardial effusion, and vascular abnormalities but has limited utility in assessing myocardial function. Cardiac MRI is the gold standard for evaluating myocardial fibrosis, oedema, and tissue characterization, making it an excellent tool for diagnosing myocardial contusion and stress cardiomyopathy. However, its limited availability, high cost, and prolonged scan times make it impractical for use in the acute trauma setting.

The limitations of traditional diagnostic methods highlight the need for a more comprehensive, multi-modal approach to assessing trauma-related cardiac dysfunction. Integrating advanced imaging techniques such as strain imaging, cardiac MRI, and artificial intelligence-driven diagnostics may improve the early detection and management of myocardial impairment. Additionally, the use of inflammatory and oxidative stress biomarkers, such as interleukin-6 (IL-6), tumor necrosis factor-alpha (TNF-α), myeloperoxidase (MPO), and intercellular adhesion molecule-1 (ICAM-1), could enhance risk stratification and provide a more detailed understanding of trauma-induced cardiac injury.

Moving forward, the development of personalized diagnostic strategies that incorporate both functional and molecular assessments will be essential in optimizing trauma care. By combining traditional diagnostic tools with emerging technologies, clinicians can improve the accuracy of cardiac assessments, facilitate early intervention, and reduce the long-term cardiovascular burden in trauma survivors. The integration of artificial intelligence and machine learning in trauma cardiology could further enhance the predictive value of diagnostic models by analyzing complex data patterns and identifying high-risk patients more effectively.

In conclusion, while traditional diagnostic methods such as ECG, conventional echocardiography, and cardiac biomarkers remain valuable in the evaluation of trauma-related cardiac dysfunction, they have significant limitations in detecting early and subclinical myocardial impairment. More advanced techniques, including speckle-tracking echocardiography, biomarker profiling, and cardiac MRI, offer greater sensitivity and specificity in assessing myocardial injury but are not yet widely implemented in trauma care. A multi-disciplinary approach that incorporates emerging diagnostic technologies, personalized risk assessment, and advanced imaging modalities is essential to improving outcomes for trauma patients with cardiac involvement. Continued research into novel diagnostic strategies and the integration of artificial intelligence in trauma cardiology will be crucial in addressing the current limitations and enhancing the precision of post-trauma cardiac evaluations.

**Table 1:** Traditional and emerging diagnostic approaches in trauma-related cardiac dysfunction.

Diagnostic Modality	Clinical Role	Limitations in Trauma Context	Emerging Enhancements/Solutions	Ref.
Electrocardiography (ECG)	Rapid bedside assessment; identifies arrhythmias, ischemia, and conduction abnormalities.	Low sensitivity for subtle myocardial injury. Non-specific changes such as transient ST-segment or T-wave abnormalities. May be normal despite significant dysfunction.	AI-enhanced ECG interpretation. Combined use with biomarkers and imaging for improved diagnostic yield.	[58,59]
Conventional Echocardiography	Assesses systolic function, wall motion, and pericardial effusion. Detects cardiac tamponade and contusion.	Left ventricular ejection fraction (LVEF) may be preserved despite dysfunction. Poor sensitivity to early or subclinical myocardial changes. Limited insight into myocardial mechanics.	Speckle-tracking echocardiography (STE) and global longitudinal strain (GLS) for early detection of myocardial dysfunction.	[60,61]
Cardiac Biomarkers (e.g., Troponin, hs-Tn)	Detects myocardial cell injury. Commonly used to evaluate myocardial infarction or contusion.	Elevated levels may result from non-cardiac trauma-related stress (inflammation, hypoxia). May be normal in cases of functional rather than structural injury. Lack of anatomical or functional context.	Multiparametric biomarker panels including IL-6, TNF-α, MPO, and ICAM-1. Use in combination with imaging and clinical scoring systems.	[62,63]
Natriuretic Peptides (BNP, NT-proBNP)	Reflects ventricular wall stress. Useful in evaluating potential heart failure.	Elevated in non-cardiac conditions such as renal dysfunction, sepsis, or volume overload. Low specificity for direct cardiac injury.	Adjunctive role in multimodal risk stratification. Should not be used as standalone diagnostics.	[64,65]
Computed Tomography (CT Angiography)	Visualizes vascular trauma, aortic injury, and pericardial effusion.	Limited capability in assessing myocardial function. Exposure to radiation. Not suitable for dynamic or functional cardiac evaluation.	Used primarily for vascular and structural assessment. Limited utility in evaluating myocardial performance.	[66,67]
Cardiac Magnetic Resonance Imaging (MRI)	High-resolution myocardial tissue characterization. Differentiates fibrosis, oedema, and inflammation.	Limited availability and high cost. Long scan times. Not practical in acute trauma settings.	Selective use in stable or subacute trauma patients. Valuable for follow-up and definitive tissue-level assessment.	[68,69]
Overall Strategy and Future Direction	Foundational role in initial cardiac assessment and Triage.	No single modality adequately captures structural, functional, and molecular abnormalities. Risk of delayed or missed diagnosis of subclinical dysfunction.	Multimodal diagnostic strategies integrating ECG, strain imaging, biomarkers, and MRI. Use of AI for risk stratification and predictive analytics. Personalized diagnostics based on trauma severity comorbidities and resource availability.	[70,71]

## 4. Longitudinal Myocardial Deformation: A Sensitive Marker for Post-Traumatic Cardiac Dysfunction

In post-traumatic cardiac dysfunction, myocardial deformation abnormalities may arise due to a combination of direct mechanical injury, systemic inflammation, oxidative stress, and neurohormonal dysregulation. Trauma patients who sustain blunt chest injuries, such as those caused by motor vehicle accidents or falls, may experience myocardial contusion, leading to localized wall motion abnormalities that can be detected using strain imaging [72,73]. However, even in cases where no overt structural damage is evident, myocardial strain analysis can reveal impairments in contractility that are not apparent on standard echocardiography.

Longitudinal myocardial deformation has emerged as a highly sensitive marker for detecting post-traumatic cardiac dysfunction, offering a more precise and quantitative assessment of myocardial mechanics than traditional diagnostic measures [74] (Table 2). Unlike conventional echocardiographic parameters, such as left ventricular ejection fraction (LVEF), which primarily reflect global systolic function, longitudinal strain provides detailed insights into myocardial contractility at the fiber level [75]. This distinction is particularly important in trauma patients, where subtle myocardial dysfunction often remains undetected using standard assessment tools. Speckle-tracking echocardiography (STE) enables clinicians to assess myocardial deformation by tracking the movement of naturally occurring acoustic markers within the myocardium throughout the cardiac cycle [76]. By analyzing the degree of myocardial shortening during systole, STE-derived GLS provides a quantitative measure of myocardial function, making it a valuable tool for early diagnosis, risk stratification, and management of trauma-induced cardiac dysfunction.

One of the key advantages of GLS over LVEF is its ability to detect myocardial dysfunction before significant changes in ejection fraction occur [77]. LVEF, while commonly used in clinical practice, is a relatively crude measure of ventricular function that can remain preserved even in the presence of significant myocardial impairment (Table 2). This phenomenon is particularly relevant in trauma patients, where compensatory mechanisms may temporarily maintain LVEF despite underlying myocardial injury. GLS, on the other hand, is more sensitive to early contractile dysfunction, allowing for the detection of subtle changes in myocardial mechanics that precede overt cardiac dysfunction [78]. Studies have shown that reduced GLS is associated with worse clinical outcomes, including increased risk of heart failure, arrhythmias, and mortality, underscoring its prognostic value in trauma-related cardiac dysfunction.

Some individuals may experience transient myocardial stunning, a reversible condition characterized by temporary contractile dysfunction due to excessive catecholamine release and ischemia–reperfusion injury. In such cases, GLS can help distinguish between reversible myocardial dysfunction and more severe forms of cardiac injury that may require long-term management. Additionally, in patients with systemic inflammatory responses following trauma, GLS can serve as an indicator of inflammation-related myocardial impairment, offering a potential link between immune activation and cardiac dysfunction.

Incorporating GLS into trauma care has the potential to improve early detection and guide targeted interventions aimed at preventing long-term complications [79]. Trauma patients with reduced GLS, even in the absence of significant LVEF reduction, may benefit from closer cardiac monitoring and early initiation of cardioprotective therapies. In particular, beta-blockers and angiotensin-converting enzyme (ACE) inhibitors have been shown to improve myocardial strain and reduce the risk of adverse cardiovascular events in patients with subclinical myocardial dysfunction. Additionally, GLS may be useful in assessing the effectiveness of anti-inflammatory and antioxidant therapies in trauma patients, providing a non-invasive means of tracking myocardial recovery over time [80,81].

The prognostic significance of GLS extends beyond acute trauma care, with implications for long-term cardiovascular health [79,82]. Trauma patients with persistently reduced GLS are at higher risk for developing chronic cardiac conditions, including heart failure and arrhythmias. As such, incorporating longitudinal strain analysis into follow-up assessments can help identify individuals who may require long-term cardiac care. Serial GLS measurements can provide valuable information on myocardial recovery, allowing clinicians to tailor treatment strategies based on individual patient trajectories. By identifying patients at risk for long-term cardiovascular complications, GLS can play a crucial role in improving long-term outcomes and reducing the burden of post-traumatic cardiac disease.

While GLS has shown significant promise in the evaluation of post-traumatic cardiac dysfunction, its widespread adoption in trauma settings faces several challenges. One limitation is the need for specialized imaging equipment and expertise in strain analysis, which may not be readily available in all healthcare settings. Additionally, interobserver variability and differences in vendor-specific strain measurement algorithms can impact the reproducibility of GLS results. Standardization of strain imaging protocols and the development of automated strain analysis tools could help address these challenges and facilitate the broader implementation of GLS in trauma care.

Future research should focus on refining the clinical applications of GLS in trauma-related cardiac dysfunction, including the establishment of standardized GLS cut-off values for identifying high-risk patients. Large-scale prospective studies are needed to validate the prognostic utility of GLS in trauma populations and determine its role in guiding therapeutic interventions. Additionally, the integration of GLS with other emerging biomarkers, such as inflammatory cytokines and oxidative stress markers, may enhance risk stratification and provide a more comprehensive understanding of the mechanisms underlying post-traumatic myocardial dysfunction.

**Table 2:** Longitudinal myocardial deformation in trauma-related cardiac dysfunction: a multidimensional overview.

Component	Description	Clinical Relevance	Advantages Over Traditional Methods	Limitations/Challenges	Future Perspectives	Ref.
Global Longitudinal Strain (GLS)	Quantitative measure of myocardial fiber shortening during systole, assessed via speckle-tracking echocardiography (STE)	Detects subclinical dysfunction in trauma patients, even with normal LVEF	Higher sensitivity to early dysfunction; fiber-level analysis	Requires advanced echocardiographic equipment and trained operators	Standardization and integration into trauma protocols	[83,84]
Mechanisms of Dysfunction	Includes direct injury, inflammation, oxidative stress, neuro- hormonal imbalance	Identifies myocardial injury even without visible structural damage	Can localize injury patterns undetectable by ECG or LVEF	Cannot differentiate exact ethology (e.g., contusion vs. inflammation) without adjunct biomarkers	Combined use with cardiac biomarkers and imaging (e.g., MRI)	[85,86]
Early Detection	Identifies early myocardial impairment prior to LVEF drop	Enables prompt intervention and monitoring	Better than conventional echocardiography for initial trauma screening	Limited access in emergency trauma settings	Implementation of point-of-care STE platforms	[87,88]
Risk Stratification	Differentiates high- from low-risk patients based on GLS thresholds	Guides intensity of monitoring and therapy	Provides prognostic information beyond ECG or biomarkers	No universally accepted GLS cut-offs in trauma	Development of trauma-specific GLS algorithms	[89,90]
Prognostic Value	Reduced GLS predicts worse outcomes (HF, arrhythmias, mortality)	Long-term risk assessment and patient counseling	Tracks recovery or deterioration longitudinally	Long-term data still limited in trauma populations	Prospective multicenter cohort validation	[91,92]
Therapeutic Monitoring	Monitors response to cardioprotective (e.g., beta-blockers) or anti-inflammatory therapies	Tailors treatment to myocardial recovery trajectory	Non-invasive and repeatable compared to cardiac MRI	Requires repeat echocardiographic access	Integration with biomarker panels and AI for monitoring	[93,94]
Versus LVEF	GLS detects dysfunction with preserved LVEF	Identifies hidden myocardial damage early	Adds value where LVEF is insensitive	Requires familiarity with strain analysis	Promote educational training for clinicians	[95,96]
Versus Biomarkers (e.g., Troponin, BNP)	GLS reflects mechanical function, while biomarkers reflect biochemical stress	Useful where biomarker levels are normal despite dysfunction	More specific to mechanical impairment	Biomarkers remain useful for systemic context	Combine GLS with inflammatory and oxidative stress markers	[97,98]

As the field of trauma cardiology continues to evolve, longitudinal myocardial deformation analysis represents a promising tool for improving the diagnosis and management of trauma-induced cardiac dysfunction. By providing a more sensitive and quantitative assessment of myocardial function, GLS has the potential to bridge the gap between early myocardial injury detection and long-term cardiovascular risk reduction. The incorporation of strain imaging into routine trauma care could lead to earlier interventions, improved patient outcomes, and a more personalized approach to post-traumatic cardiac management. With continued advancements in imaging technology and a growing body of clinical evidence supporting its use, GLS is poised to become an essential component of trauma cardiology, helping to redefine how myocardial dysfunction is detected and treated in trauma patients.

## 5. Clinical Applications of Longitudinal Myocardial Deformation in Trauma Care

Longitudinal myocardial deformation has become a valuable tool for assessing heart function in trauma patients, offering greater sensitivity than traditional methods. Unlike conventional measures such as left ventricular ejection fraction (LVEF), which may miss early signs of cardiac impairment, this technique provides detailed insights into trauma-related myocardial dysfunction [99,100]. Its clinical uses range from early detection and risk assessment to guiding treatment and monitoring long-term heart health. A key advantage is its ability to identify myocardial dysfunction early in patients with blunt chest trauma, such as those from car accidents or falls, where injuries may not be apparent on standard imaging or ECG.

Speckle-tracking echocardiography (STE), which measures GLS, can detect subtle changes in heart muscle contractility even when LVEF appears normal [88]. This makes GLS a highly effective tool for diagnosing and managing cardiac injury in trauma care (Table 3).

In addition to its role in detecting myocardial contusion, longitudinal myocardial deformation is particularly useful in assessing myocardial dysfunction in patients with systemic inflammatory responses following severe trauma [101]. Trauma induces a cascade of inflammatory and oxidative stress responses that can lead to myocardial stunning and impaired contractility. Elevated levels of inflammatory cytokines, such as interleukin-6 (IL-6) and tumor necrosis factor-alpha (TNF-α), have been associated with myocardial dysfunction in trauma patients, contributing to contractile impairment and ventricular remodeling [102]. GLS provides a quantitative assessment of myocardial function that correlates with the extent of inflammatory-mediated myocardial injury, allowing clinicians to better understand the interplay between systemic inflammation and cardiac dysfunction [103]. By integrating GLS with biomarker analysis, clinicians can more accurately assess the severity of trauma-induced myocardial dysfunction and tailor treatment strategies accordingly (Table 3).

Another critical application of longitudinal myocardial deformation in trauma care is its use in risk stratification and prognostication (Table 3). Trauma patients who exhibit reduced GLS, even in the absence of overt cardiac symptoms, may be at increased risk for developing long-term cardiovascular complications, such as heart failure and arrhythmias. The ability to identify high-risk patients early in their clinical course enables the implementation of more aggressive monitoring and therapeutic interventions aimed at preventing disease progression. Studies have demonstrated that GLS abnormalities are predictive of adverse cardiovascular events in various clinical settings, including heart failure and myocardial infarction, suggesting that similar principles may apply in trauma populations [38,103,104]. In this context, GLS could serve as a valuable risk assessment tool to guide clinical decision-making and improve patient outcomes.

Beyond risk stratification, longitudinal myocardial deformation plays a crucial role in guiding therapeutic interventions in trauma patients with cardiac dysfunction. The ability to detect early myocardial impairment allows for timely initiation of cardioprotective therapies, such as beta-blockers and angiotensin-converting enzyme (ACE) inhibitors, which have been shown to improve myocardial function and reduce the risk of long-term complications [105]. Patients with evidence of strain abnormalities may benefit from closer hemodynamic monitoring, fluid optimization, and early intervention to prevent the development of heart failure. Furthermore, GLS can be used to evaluate the efficacy of anti-inflammatory and antioxidant therapies in trauma patients, providing a non-invasive means of assessing treatment response and myocardial recovery.

The role of longitudinal myocardial deformation extends beyond the acute phase of trauma care, with significant implications for long-term cardiovascular monitoring [106]. Trauma patients who survive their initial injuries may continue to experience subclinical myocardial dysfunction that places them at risk for developing chronic cardiac conditions [107]. GLS can be utilized as a longitudinal monitoring tool to track myocardial function over time and assess the trajectory of cardiac recovery. Serial GLS measurements can provide valuable information on myocardial remodeling and help determine whether patients require ongoing cardiac management. This is particularly important for patients with persistent strain abnormalities as they may benefit from continued medical therapy and lifestyle modifications to mitigate the risk of future cardiovascular events.

Another promising application of longitudinal myocardial deformation in trauma care is its integration with other advanced imaging modalities, such as cardiac magnetic resonance imaging (MRI) and computed tomography (CT) [108]. While GLS provides detailed insights into myocardial mechanics, cardiac MRI offers superior tissue characterization, allowing for the detection of myocardial fibrosis, oedema, and scarring [109]. Combining these modalities could enhance diagnostic accuracy and provide a more comprehensive assessment of trauma-induced myocardial injury. Additionally, the integration of artificial intelligence and machine learning algorithms into strain imaging analysis holds the potential to further refine risk prediction models and improve the clinical utility of GLS in trauma populations.

**Table 3:** Clinical applications of longitudinal myocardial deformation in trauma care.

Clinical Application	Description	Key Benefits	Challenges	Ref.
Early Detection of Cardiac Injury	Detects subclinical myocardial dysfunction in blunt chest trauma, even when ECG and LVEF are normal.	Enables early diagnosis and intervention for myocardial contusion or trauma-induced contractility impairment.	May be overlooked in patients without visible trauma; requires high-resolution imaging and trained operators.	[110,111]
Assessment During Systemic Inflammatory Response	Evaluates myocardial dysfunction linked to inflammation and oxidative stress post-trauma (e.g., IL-6, TNF-α).	Provides insight into inflammation-induced cardiac injury and helps guide anti-inflammatory treatment.	Requires biomarker correlation; inflammation-related changes may be transient or confounded by other injuries.	[112,113]
Risk Stratification and Prognostication	Identifies high-risk patients with reduced GLS for future cardiac complications, even if asymptomatic.	Supports targeted monitoring and early therapeutic strategies to prevent heart failure and arrhythmias.	No established trauma-specific GLS thresholds; prognostic implications need further validation.	[114,115]
Guiding Therapeutic Interventions	Informs timely initiation of cardioprotective therapies (e.g., beta-blockers, ACE inhibitors) and fluid optimization.	Improves outcomes by tailoring treatments to individual myocardial function.	Requires dynamic monitoring and clinical interpretation; risk of over-treatment in ambiguous cases.	[116]
Long-Term Cardiac Monitoring	Tracks myocardial recovery and remodeling in trauma survivors through serial GLS measurements.	Enables personalized follow-up and long-term care planning.	Long-term access to imaging may be limited; adherence to follow-up may be poor.	[117]
Integration with Advanced Imaging Modalities	Combines with cardiac MRI/CT to assess structural damage (e.g., fibrosis, oedema, scarring).	Enhances diagnostic accuracy and understanding of myocardial pathology.	MRI/CT access may be limited; high cost and patient instability may preclude use in acute trauma.	[118,119]
AI and Machine Learning Integration	Enhances GLS analysis and risk prediction through automated algorithms and data-driven models.	Improves consistency, speed, and predictive power of strain interpretation.	Requires robust datasets; AI systems must be validated for trauma populations.	[120]
Implementation in Clinical Practice	Offers a non-invasive, sensitive tool for routine trauma cardiac assessment.	Facilitates evidence-based, personalized trauma cardiology care.	Requires specialized echocardiography equipment and training; vendor variability in GLS measurements.	[38]
Future Research and Validation	Needed for establishing trauma-specific GLS reference values and studying utility in subgroups (e.g., TBI, hemorrhagic shock).	Expands clinical relevance and optimizes use in diverse trauma settings.	Limited large-scale prospective studies; subgroup-specific data currently sparse.	[121]

Despite its numerous advantages, the widespread adoption of longitudinal myocardial deformation in trauma care faces several challenges. One of the primary limitations is the need for specialized imaging equipment and expertise in strain analysis, which may not be readily available in all healthcare settings. Additionally, variability in strain measurement techniques between different echocardiographic vendors can impact the reproducibility of GLS results, highlighting the need for standardized protocols and calibration methods. Efforts to address these challenges through training programs, standardized guidelines, and automated strain analysis tools will be critical in facilitating the broader implementation of GLS in trauma care.

While speckle tracking echocardiography (STE) offers promising sensitivity for detecting subclinical myocardial dysfunction, its broader clinical application is limited by several important factors. First, the accuracy of strain measurements is highly dependent on the operator’s experience, both in image acquisition and post-processing interpretation. Second, the quality of the echocardiographic images themselves, including endocardial border definition, plays a critical role, particularly in trauma settings where imaging conditions may be suboptimal. Third, frame rate settings must be carefully optimized as strain analysis can be significantly affected by excessively low or high temporal resolution. Fourth, STE is sensitive to changes in loading conditions (preload and afterload), which can alter myocardial deformation patterns independent of intrinsic cardiac function. Fifth, extrinsic mechanical factors, especially chest wall conformation and soft tissue variability, can degrade image quality and strain reliability, particularly in patients with obesity, chest trauma, or surgical dressings [122,123]. Finally, it is important to note that STE remains underutilized in daily clinical practice due to barriers such as limited training, a lack of familiarity among general practitioners, and time constraints in emergency and ICU environments [124]. These limitations underscore the need for standardized protocols, automated software tools, and broader clinician education to enable more consistent use of STE in trauma care settings.

Future research should focus on further validating the clinical applications of longitudinal myocardial deformation in trauma populations through large-scale prospective studies. Establishing GLS reference values for trauma patients and determining optimal cut-off thresholds for risk stratification will enhance its utility as a clinical tool. Additionally, exploring the role of GLS in specific trauma subgroups, such as patients with traumatic brain injury or hemorrhagic shock, could provide valuable insights into its broader applicability in critical care settings. As the field of trauma cardiology continues to evolve, longitudinal myocardial deformation is poised to play an increasingly important role in improving the diagnosis, management, and long-term outcomes of patients with trauma-induced cardiac dysfunction.

The ability of GLS to detect early myocardial dysfunction, guide treatment decisions, and predict long-term cardiovascular risk makes it a highly valuable tool in trauma care. By incorporating strain imaging into routine clinical practice, clinicians can achieve a more comprehensive understanding of myocardial function and provide more personalized, evidence-based care for trauma patients. With continued advancements in imaging technology and increasing recognition of the importance of myocardial strain analysis, longitudinal myocardial deformation is set to become a cornerstone of trauma cardiology, paving the way for improved patient outcomes and more effective management of post-traumatic cardiac dysfunction.

## 6. Future Directions and Research Opportunities

Future directions and research opportunities in the field of longitudinal myocardial deformation and post-traumatic cardiac dysfunction are vast, offering significant potential to improve early diagnosis, risk stratification, and patient outcomes. As the understanding of trauma-induced myocardial dysfunction continues to evolve, there is a growing need for interdisciplinary approaches that integrate advanced imaging techniques, biomarker analysis, and artificial intelligence-driven diagnostics. Expanding research in these areas will enhance clinicians’ ability to detect myocardial dysfunction at its earliest stages, refine therapeutic strategies, and develop personalized treatment plans that reduce long-term cardiovascular risk in trauma patients.

One of the most promising areas for future research is the optimization and standardization of GLS measurement in trauma populations. While GLS has demonstrated superior sensitivity in detecting subclinical myocardial dysfunction compared to traditional metrics such as left ventricular ejection fraction (LVEF), significant variability remains in strain measurement techniques across different imaging platforms and vendors [125,126]. Standardizing GLS protocols and establishing trauma-specific reference values will be essential to improving its clinical utility. Large-scale, multi-center studies should be conducted to define normative GLS ranges in trauma patients and determine the optimal cut-off values for identifying individuals at risk for adverse cardiac outcomes. Additionally, further investigation into the correlation between GLS abnormalities and long-term cardiovascular events in trauma survivors will help refine risk prediction models and inform clinical decision-making.

The integration of GLS with emerging cardiac biomarkers represents another exciting avenue for future research. While strain imaging provides valuable mechanical insights into myocardial function, the addition of biomarker profiling could offer a more comprehensive assessment of trauma-induced cardiac dysfunction. Inflammatory mediators such as interleukin-6 (IL-6), tumor necrosis factor-alpha (TNF-α), and interleukin-1 beta (IL-1β) have been shown to play critical roles in post-traumatic myocardial impairment, and their relationship with myocardial strain abnormalities warrants further investigation [127]. Additionally, oxidative stress markers, including myeloperoxidase (MPO) and reactive oxygen species (ROS), may provide complementary information on the pathophysiological mechanisms underlying myocardial dysfunction in trauma patients. Future research should explore the potential of combining GLS with biomarker panels to create multi-modal risk assessment tools that enhance early detection and treatment strategies.

Artificial intelligence (AI) and machine learning have the potential to revolutionize the application of longitudinal myocardial deformation analysis in trauma care [128,129]. AI-driven algorithms can be trained to detect subtle myocardial strain abnormalities with greater accuracy and reproducibility, reducing operator-dependent variability in GLS measurements. Machine learning models could also integrate data from echocardiographic imaging, biomarker profiling, and electronic health records to predict which trauma patients are at the highest risk for developing long-term cardiovascular complications [129]. Future research should focus on developing AI-assisted diagnostic platforms that leverage big data to enhance the clinical interpretation of myocardial strain analysis and improve patient outcomes.

The role of longitudinal myocardial deformation in guiding therapeutic interventions also warrants further exploration [130]. While GLS has primarily been used as a diagnostic and prognostic tool, its potential as a treatment-monitoring parameter remains largely unexplored. Future studies should investigate whether serial GLS measurements can be used to track the efficacy of cardioprotective therapies in trauma patients. For example, beta-blockers, angiotensin-converting enzyme (ACE) inhibitors, and anti-inflammatory agents have shown promise in improving myocardial function in other cardiac conditions, but their impact on myocardial strain recovery in trauma patients remains unknown. Conducting clinical trials that evaluate how GLS responds to specific pharmacologic interventions could help establish evidence-based treatment guidelines for post-traumatic cardiac dysfunction.

Longitudinal studies assessing the long-term cardiovascular impact of trauma-related myocardial dysfunction are critically needed. Many trauma patients, even those who appear to recover fully, may experience lingering cardiac effects that predispose them to heart failure, arrhythmias, and ischemic heart disease later in life. Prospective cohort studies following trauma patients over extended periods will help clarify the relationship between early GLS abnormalities and long-term cardiovascular risk. Additionally, identifying subgroups of trauma patients who are particularly susceptible to chronic cardiac complications—such as those with pre-existing cardiovascular conditions, systemic inflammatory responses, or prolonged ICU stays—will enable the development of targeted monitoring and intervention strategies.

Advancing the role of GLS in pediatric trauma populations is another important area for future research. While myocardial strain imaging has been extensively studied in adults, there are limited data on its application in pediatric trauma patients. Children and adolescents may exhibit different myocardial adaptation responses following trauma, necessitating age-specific GLS reference values and diagnostic criteria. Research efforts should focus on evaluating how longitudinal myocardial deformation evolves across different pediatric age groups and determining whether early GLS abnormalities in children correlate with long-term cardiovascular consequences.

The exploration of novel therapeutic targets for mitigating trauma-induced myocardial dysfunction is another crucial direction for future research. Beyond conventional pharmacologic interventions, regenerative medicine approaches, such as stem cell therapy and tissue engineering, hold promise for repairing myocardial damage following trauma. Preclinical studies suggest that mesenchymal stem cells (MSCs) and exosome-based therapies may enhance myocardial regeneration and improve cardiac function in animal models of injury [131]. Translating these findings into clinical practice will require rigorous clinical trials to assess the safety and efficacy of regenerative therapies in trauma patients.

Finally, research should focus on improving the accessibility and implementation of strain imaging in diverse healthcare settings. While GLS is a powerful diagnostic tool, its widespread adoption is hindered by equipment limitations, training requirements, and cost barriers. Efforts to develop more user-friendly, automated strain imaging technologies could help expand the availability of GLS in both high-resource and low-resource settings. Additionally, integrating GLS into trauma care protocols and emergency medicine workflows will be essential for ensuring its timely use in high-acuity settings.

**Figure 1:**
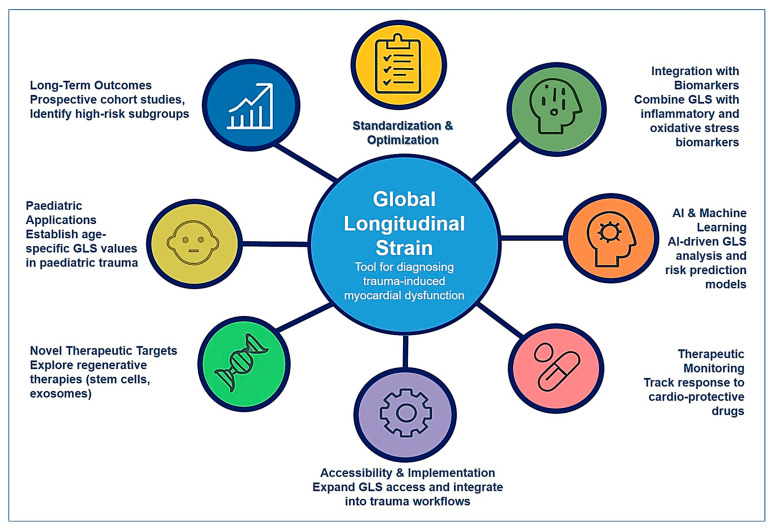
Future directions and research opportunities in longitudinal myocardial deformation (GLS) in trauma care.

Future directions and research opportunities in the field of longitudinal myocardial deformation and trauma-related cardiac dysfunction hold immense potential to transform patient care (Figure 1). By refining diagnostic methodologies, integrating biomarker-based risk assessment, leveraging artificial intelligence, exploring novel therapies, and conducting long-term follow-up studies, researchers can advance the understanding and management of trauma-induced myocardial dysfunction. As these advancements continue to unfold, the clinical application of GLS is likely to become an integral component of trauma cardiology, leading to earlier detection, improved treatment strategies, and better long-term cardiovascular outcomes for trauma patients.

## 7. Conclusions

Longitudinal myocardial deformation, assessed through global longitudinal strain (GLS), represents a significant advancement in the evaluation of cardiac function following trauma. Unlike traditional diagnostic tools such as electrocardiography, left ventricular ejection fraction (LVEF), or isolated biomarker measurements, GLS offers a sensitive and quantitative measure of subclinical myocardial impairment that often remains undetected in the acute trauma setting. Its ability to identify early contractile dysfunction before structural or hemodynamic deterioration occurs makes it an invaluable tool for timely risk stratification and clinical decision-making.

This review underscores the complex, multifactorial nature of post-traumatic cardiac dysfunction, driven by direct mechanical injury, systemic inflammation, oxidative stress, and neuro-hormonal activation. GLS has demonstrated clinical relevance across the entire care continuum, from early detection and prognostication to monitoring treatment response and guiding long-term cardiovascular follow-up. Furthermore, the integration of GLS with inflammatory and oxidative stress biomarkers, along with artificial intelligence-driven analytics, holds promise for developing a precision medicine framework in trauma cardiology.

Despite the growing interest in global longitudinal strain (GLS) as a sensitive and non-invasive marker for post-traumatic myocardial dysfunction, several limitations currently constrain its widespread clinical adoption in trauma settings. First, most of the available data regarding GLS in trauma patients are derived from small-scale, single-center observational studies with limited generalizability. There remains a lack of large, prospective, multicenter trials specifically focused on trauma populations, which hampers the development of standardized GLS-based diagnostic or prognostic algorithms in this context. Second, technical variability in image acquisition protocols and post-processing algorithms used for strain analysis, such as differences between vendor-specific software and inter-observer variability, introduces challenges in reproducibility and cross-institutional consistency. These issues are particularly relevant in acute trauma care, where echocardiographic imaging conditions may be suboptimal. Addressing these limitations through standardized protocols and multicenter validation efforts will be essential to fully integrate GLS into trauma care pathways.

In conclusion, GLS has the potential to redefine the diagnostic and prognostic landscape of trauma-induced cardiac dysfunction. By enabling earlier detection, more accurate risk assessment, and individualized patient management, longitudinal myocardial deformation may significantly improve outcomes for trauma patients and reduce the long-term burden of cardiovascular complications.

## Data Availability

Not applicable.
